# Isolation, morphological identification and *in vitro* antibacterial activity of endophytic bacteria isolated from *Azadirachta indica* (neem) leaves

**DOI:** 10.14202/vetworld.2017.510-516

**Published:** 2017-05-11

**Authors:** Ankit Kumar Singh, Rajesh Kumar Sharma, Varsha Sharma, Tanmay Singh, Rajesh Kumar, Dimple Kumari

**Affiliations:** 1 Department of Veterinary Pharmacology and Toxicology, College of Veterinary Science and Animal Husbandry, Nanaji Deshmukh Veterinary Science University, Jabalpur - 482 001, Madhya Pradesh, India; 2 Department of Veterinary Microbiology, College of Veterinary Science and Animal Husbandry, Nanaji Deshmukh Veterinary Science University, Jabalpur - 482 001, Madhya Pradesh, India; 3 Department of Animal Nutrition, College of Veterinary Science and Animal Husbandry, Nanaji Deshmukh Veterinary Science University, Jabalpur - 482 001, Madhya Pradesh, India; 4 Department of Veterinary Biotechnology, College of Veterinary Science and Animal Husbandry, Nanaji Deshmukh Veterinary Science University, Jabalpur - 482 001, Madhya Pradesh, India

**Keywords:** antibacterial activity, *Azadirachta indica* (neem), ciprofloxacin, endophytic bacteria, leaves

## Abstract

**Aim:**

The objective of this study was to isolate endophytic bacteria from Azadirachta indica (neem) leaves, their identification and investigate their antibacterial activity against three Gram-positive bacteria, Staphylococcus aureus, Streptococcus pyogenes and Bacillus cereus and Gram-negative bacteria Escherichia coli, Salmonella Typhimurium and Klebsiella pneumoniae.

**Materials and Methods:**

Fresh leaves of A. indica (neem) was procured from the Department of Botany, JNKVV, Jabalpur. Five samples were taken, and each sample was divided into five subsamples and separated for further isolation of endophytic bacteria. For sterilization leaves were treated with double distilled water, 0.1% sodium hypochlorite, 0.01% bavistin, 0.05% and 70% ethanol. Sterilized leaves of the plants were embedded in Kings B (KB) petri plates and incubated at 37°C for 24 h. Characterization of the bacteria was done according to its morphology and by Gram-staining. After that, a single colony was transferred into brain heart infusion (BHI) broth and incubated at 37°C for 24 h. The antibacterial effect was studied by the disk diffusion method with known antibiotic ciprofloxacin (Ci) as standard.

**Results:**

A total of 25 bacterial isolates from A. indica (neem) were obtained and identified morphologically. Most of the samples on KB media depicted irregular shape, flat elevation, undulated, rough, opaque, and white in color. Most of the samples on blood agar showed irregular, raise elevation, undulated, smooth, opaque and all the isolates were nonhemolytic and nonchromogenic. The growth of endophytic bacteria in BHI broth were all isolates showed turbidity. The microscopic examination revealed that maximum isolates were Gram-positive and rod shaped. Good antibacterial activity was observed against S. aureus, Streptococcus pyogenes, E. coli, Salmonella Typhimurium, and K. pneumoniae.

**Conclusions:**

Endophytic bacteria are present in leaves of A. indica (neem) and it possesses antibacterial activity against few Gram-positive and Gram-negative bacteria.

## Introduction

An increase in the number of people in the world, having health problems leading to various types of cancers, drug-resistant bacteria, parasitic protozoans and fungal infection is a cause for alarm. An intensive search for newer and more effective agents to deal with these disease problems is now underway and endophytes are a novel source of potentially useful medicinal compounds.

Endophytes are microorganisms, including bacteria that live in the intercellular spaces of plant without showing any disease symptoms to the host plant [1]. Many studies have emphasized endophytes from medicinal plants and their application in different areas [2]. Recently, many known, as well as new endophytic bioactive metabolites, possessing a wide variety of biological activities as antibiotic, antiviral, anticancer, anti-inflammatory, antioxidant, etc., have been identified [3].

*Azadirachta indica* (neem) is a divine tree mainly cultivated in the Indian subcontinent, belonging to the botanical family *Meliaceae*, commonly known as neem [4]. All the parts of *A. indica* tree are commonly used in traditional Indian medicine for household remedy against various human diseases. In India, various parts of neem (leaf, bark, and seeds) are used as a traditional medicine. Different parts of neem have been shown to exhibit wide pharmacological activities such as antibacterial [5], antiviral [6], anti-inflammatory activity [7], anti-carcinogenic activity [8], parasitic disease [9], and skin disease [8].

The objective of this study was to isolate endophytic bacteria from neem leaves, their identification and investigate their antibacterial activity against three Gram-positive bacteria, *Staphylococcus aureus*, *Streptococcus pyogenes* and *Bacillus cereus* and Gram-negative bacteria *Escherichia coli*, *Salmonella* Typhimurium, and *Klebsiella pneumoniae*.

## Materials and Methods

### Ethical approval

In this work, no animals were used therefore there was no requirement of ethical approval.

### Plant material

Fresh leaves of *A. indica* (neem) were procured from the Department of Botany, JNKVV, Jabalpur. Fresh, healthy plant leaves were collected by selected medicinal plants, viz., *A. indica* (neem). Five samples from each tree were taken and each sample was divided into five subsamples and separated for further isolation of endophytic bacteria. Samples were immediately brought to the laboratory and were used within 24 h and finally processed for isolation of endophytic bacteria.

### Sterilization of leaves

The sterilization of leaves and isolation of endophytic bacteria from the leaves was done according to Mahajan *et al*. [10], with some modifications. The leaves were treated with double distilled water for 2-3 min, then surface sterilized with 0.1% sodium hypochlorite for 5 min, washed with double distilled water for 2-3 min. Later, surface sterilization was done with 0.01% bavistin and was kept in distilled water for 5 min. For further sterilization, the leaves were exposed to 0.05% streptomycin followed by treatment with double distilled water for 5 min. Then, the leaves were exposed to 70% ethanol and again were kept in double distilled water for 5 min and were excised with autoclaved scalpel and forceps in the laminar air flow chamber, then air dried in laminar flow.

### Sterility check

To confirm that the surface of leaves were effectively sterilized 1 ml of the sterile distilled water that was used in the final rinse of surface sterilization procedures were planted onto nutrient agar media and incubated at 37°C for 24 h. Bacterial growths were observed after 24 h. Furthermore, surface sterilized leaves were rolled on nutrient agar plates and incubated at 37°C for 24 h and checked for possible microbial growth.

### Preparation and sterilization of media

King’s B (KB) media, Mueller–Hinton media, blood agar media, and brain heart infusion (BHI) broth were prepared by adding agar in the distilled water. Hotplate was used for the proper mixing of media and autoclaved at 121°C for 15-20 min at 15 lbs.

### Inoculation of leaves

The media was poured into different autoclaved petri plates and leaves of the plants were embedded in petri plates. These plates were then incubated at 37°C for 24 h. Characterization of the bacteria was done according to its morphology and by Gram-staining. After that, a single colony was transferred into BHI broth and incubated at 37°C for 24 h.

### Purification of endophytic bacteria

For purification of endophytic bacteria subculturing was mainly done by streaking a loop full of BHI broth on the fresh pre-solidified blood agar plates and then incubated at 37°C for 24 h. After incubation, the colonies were transferred into BHI broth and then incubated at 37°C for 24 h and purity is checked by Gram-stain and stored at −20°C in deep freeze for further work.

### Antibacterial activity of endophytic bacteria

a. *In vitro* study.

### Procurement of known culture (Table-1)

**Table-1:** List of procured bacterial culture.

Bacteria	ATCC catalogue No.
*Escherichia coli*	25,922
*Klebsiella pneumoniae*	700,603
*Salmonella* Typhimurium	13,311
*Bacillus cereus*	11,778
*Staphylococcus aureus*	6538
*Streptococcus pyogenes*	12,386

ATCC: American Type Culture Collection

#### Preparation of inoculums

The above prepared bacterial inoculums were evenly spread on sterile Mueller-Hinton agar plates as described by Bauer *et al*. [11], and antibacterial effect was studied by the disk diffusion method in these plates. The known antibiotic ciprofloxacin (Ci) was simultaneously used and placed as a control for antibiotic sensitivity. The dried disks were immediately used and incubated at 37°C for 24 h.

#### Preparation of antibacterial disk

For determination of antibacterial activity of endophytic bacteria, broths was centrifuged at 4°C at 12,000 rpm for 30 min. Supernatant of each of these broths was taken, sterile disks were soaked in these broths in sterile test tubes for 24 h and dried in laminar flow. After drying, the disks were used immediately for disk impregnation in the inoculated plates as described by Kirubaharan *et al*. [12] with slight modifications. Ci disk were used as a control drug to compare the effect of treatment during *in vitro* study.

### Antibacterial test

The prepared bacterial inoculums were evenly spread on a sterile Mueller-Hinton agar plate as per method described by Bauer *et al*. [11]. The known antibiotic Ci was simultaneously placed as a control for antibiotic sensitivity. The dried disk was incubated at 37°C for 24 h. Results were recorded as positive (growth) or negative (no growth) and zone of inhibition of growth exerted by these impregnated disks.

## Results

This study was conducted with a view to isolate and characterizes the endophytic bacterial diversity from *A. indica* (neem). 25 bacterial isolates from *A. indica* (neem) were obtained and identified morphologically. These endophytic bacteria were evaluated for *in vitro* antibacterial activity.

The sterilized leaves of *A. indica* (neem) were put in the KB media and incubated at 37°C at 24 h. The morphological characterization of endophytic bacterial isolates exhibited diverse colony shape, color margin and texture, including round to irregular colonies and white and yellow colonies with irregular and wavy margins. The endophytic bacterial isolates recovered from KB media showed soft and mucoid colonies.

### Preliminary characterization of isolated endophytic bacteria

#### Growth of endophytic bacteria in KB medium

Growth characteristics of endophytic bacteria isolated from neem leaves showed that 84% were irregular in shape while 16% circular in shape, 64% were flat elevation on petri plate while 36% raised elevation, margin of the 72% colonies were undulated while 28% entire, the surface of the growth was rough in 72% while 16% entire and 12% smooth and 96% growth were opaque and white in color (Table-2).

**Table-2:** Growth of endophytic bacteria isolated from *Azadirachta indica* (neem) on KB media.

Isolate No.	Form	Elevation	Margin	Surface	Opacity	Chromogenesis
N1a	Irregular	Flat	Undulated	Rough	Opaque	Absent
N1b	Irregular	Raised	Undulated	Rough	Opaque	Absent
N1c	Irregular	Flat	Undulated	Rough	Opaque	Absent
N1d	Circular	Flat	Entire	Dull	Opaque	Absent
N1e	Irregular	Raised	Undulated	Rough	Opaque	Absent
N2a	Irregular	Flat	Undulated	Dull	Opaque	Absent
N2b	Irregular	Raised	Undulated	Rough	Opaque	Absent
N2c	Irregular	Raised	Undulated	Smooth	Opaque	Absent
N2d	Irregular	Flat	Undulated	Rough	Opaque	Absent
N2e	Irregular	Flat	Entire	Smooth	Opaque	Absent
N3a	Irregular	Flat	Undulated	Rough	Glistening	Absent
N3b	Irregular	Flat	Undulated	Rough	Opaque	Absent
N3c	Circular	Flat	Entire	Rough	Opaque	Absent
N3d	Circular	Flat	Undulated	Smooth	Opaque	Absent
N3e	Irregular	Flat	Entire	Rough	Opaque	Green
N4a	Irregular	Raised	Undulated	Rough	Opaque	Absent
N4b	Irregular	Flat	Undulated	Rough	Opaque	Absent
N4c	Irregular	Flat	Entire	Smooth	Opaque	Absent
N4d	Irregular	Flat	Undulated	Dull	Opaque	Absent
N4e	Irregular	Flat	Undulated	Rough	Opaque	Absent
N5a	Irregular	Raised	Entire	Rough	Opaque	Absent
N5b	Irregular	Raised	Undulated	Dull	Opaque	Absent
N5c	Irregular	Flat	Entire	Rough	Opaque	Absent
N5d	Circular	Raised	Undulated	Rough	Opaque	Absent
N5e	Irregular	Flat	Undulated	Rough	Opaque	Absent

#### Growth of endophytic bacteria on 5% sheep blood agar medium

Colonies of endophytic bacteria grown on KB agar were transferred to the 5% sheep blood agar plates and incubated at 37°C for 24 h. The growth of endophytic bacteria from *A. indica* (neem) was studied.

Growth characteristics of endophytic bacteria isolated from neem leaves presented that 76% were irregular in shape while 34% circular in shape, 84% were raised elevation on petri plate while 16% flat elevation, margin of the 88% colonies were undulated while 12% entire, the surface of the growth was smooth in 92% while 8% rough; the growth was opaque in 96% isolates. All the isolates were nonhemolytic and nonchromogenic (Table-3).

**Table-3:** Growth of endophytic bacteria isolated from *Azadirachta indica* (neem) on 5% sheep blood agar.

Sample No.	Form	Elevation	Margin	Surface	Opacity	Chromogenesis
N1a	Irregular	Raised	Undulated	Smooth	Opaque	Absent
N1b	Irregular	Raised	Undulated	Smooth	Opaque	Absent
N1c	Irregular	Raised	Undulated	Smooth	Opaque	Absent
N1d	Circular	Raised	Undulated	Smooth	Opaque	Absent
N1e	Irregular	Raised	Undulated	Smooth	Opaque	Absent
N2a	Circular	Raised	Undulated	Smooth	Opaque	Absent
N2b	Irregular	Raised	Undulated	Smooth	Opaque	Absent
N2c	Irregular	Flat	Undulated	Smooth	Opaque	Absent
N2d	Irregular	Raised	Undulated	Smooth	Opaque	Absent
N2e	Irregular	Raised	Undulated	Smooth	Opaque	Absent
N3a	Circular	Raised	Undulated	Smooth	Glistening	Absent
N3b	Irregular	Raised	Undulated	Smooth	Opaque	Absent
N3c	Circular	Flat	Entire	Rough	Opaque	Absent
N3d	Circular	Raised	Undulated	Smooth	Opaque	Absent
N3e	Irregular	Raised	Undulated	Smooth	Opaque	Green
N4a	Irregular	Raised	Undulated	Smooth	Opaque	Absent
N4b	Irregular	Raised	Undulated	Smooth	Opaque	Absent
N4c	Irregular	Raised	Entire	Smooth	Opaque	Absent
N4d	Irregular	Flat	Undulated	Smooth	Opaque	Absent
N4e	Irregular	Raised	Undulated	Smooth	Opaque	Absent
N5a	Irregular	Raised	Entire	Smooth	Opaque	Absent
N5b	Irregular	Raised	Undulated	Rough	Opaque	Absent
N5c	Irregular	Raised	Undulated	Smooth	Opaque	Absent
N5d	Circular	Raised	Undulated	Smooth	Opaque	Absent
N5e	Irregular	Flat	Undulated	Smooth	Opaque	Absent

#### Growth of endophytic bacteria in BHI broth

Colonies of endophytic bacteria grown on blood agar were transferred to the sterile BHI broth tubes and incubated at 37°C for 24 h. The growth of endophytic bacteria from *A. indica* (neem).

Endophytic bacteria from neem leaves shown characteristics as 100% isolates with turbidity, 92% isolates with flocculent growth and 100% isolates with pellicle formation. No sediment formation was seen in 88% isolates and 52% isolates showed ring formation (Table-4).

**Table-4:** Growth of endophytic bacteria isolated from *Azadirachta indica* (neem) on BHI broth.

Isolate No.	Turbidity	Flocculant	Pellicle	Sediment	Ring formation
N1a	Present	Present	Present	Absent	Absent
N1b	Present	Present	Present	Absent	Absent
N1c	Present	Present	Present	Absent	Present
N1d	Present	Present	Present	Absent	Absent
N1e	Present	Absent	Present	Present	Present
N2a	Present	Present	Present	Absent	Absent
N2b	Present	Present	Present	Absent	Present
N2c	Present	Present	Present	Absent	Present
N2d	Present	Present	Present	Absent	Present
N2e	Present	Present	Present	Absent	Absent
N3a	Present	Present	Present	Absent	Absent
N3b	Present	Present	Present	Absent	Present
N3c	Present	Absent	Present	Absent	Present
N3d	Present	Present	Present	Absent	Absent
N3e	Present	Present	Present	Absent	Present
N4a	Present	Present	Present	Absent	Absent
N4b	Present	Present	Present	Present	Present
N4c	Present	Present	Present	Absent	Absent
N4d	Present	Absent	Present	Absent	Present
N4e	Present	Present	Present	Absent	Absent
N5a	Present	Present	Present	Absent	Absent
N5b	Present	Present	Present	Absent	Present
N5c	Present	Present	Present	Present	Absent
N5d	Present	Present	Present	Absent	Present
N5e	Present	Present	Present	Absent	Absent

BHI=Brain heart infusion

### Microscopic examination

The microscopic examination of endophytic bacteria was done by using Gram-stain. The results of Gram-staining are follows: 88% isolates shown Gram-positive reaction while 12% were Gram-negative, 88% endophytic bacteria were rod shape, and 12% were cocci. Microscopic examination showed that more than one type of endophytic bacteria was present in 84% of isolate (Table-5).

**Table-5:** Gram-staining of endophytic bacteria isolated from *Azadirachta indica* (neem).

Isolate No.	Gram-staining	Shape	Types of bacteria
N1a	Positive	Bacillus	<1
N1b	Positive	Bacillus	<1
N1c	Positive	Bacillus	<1
N1d	Positive	Bacillus	1
N1e	Negative	Cocci	<1
N2a	Positive	Bacillus	<1
N2b	Positive	Bacillus	<1
N2c	Positive	Bacillus	<1
N2d	Positive	Bacillus	<1
N2e	Positive	Bacillus	<1
N3a	Positive	Bacillus	1
N3b	Positive	Bacillus	<1
N3c	Positive	Bacillus	<1
N3d	Positive	Bacillus	<1
N3e	Positive	Bacillus	<1
N4a	Positive	Cocci	<1
N4b	Negative	Bacillus	<1
N4c	Positive	Bacillus	<1
N4d	Positive	Bacillus	1
N4e	Positive	Bacillus	<1
N5a	Positive	Cocci	<1
N5b	Negative	Bacillus	<1
N5c	Positive	Bacillus	<1
N5d	Positive	Bacillus	<1
N5e	Positive	Bacillus	1

### In vitro antibacterial activity

#### Antibacterial sensitivity

The antibacterial activity of endophytic bacteria was evaluated against various Gram-positive and Gram-negative pathogenic bacteria, namely, *B. cereus, S. aureus, Streptococcus pyogenes, E. coli, K. pneumoniae*, and *Salmonella* Typhimurium. Results were recorded for zone of inhibition around the disk. The inhibitory zone around the disk indicated absence of bacterial growth reported as sensitive and absence of zone reported as resistant.

#### For Gram-positive bacteria

The *in vitro* antibacterial activities of the endophytic bacteria against different Gram-positive bacteria are shown in Table-6. The endophytic bacteria isolated from *A. indica* (neem) shown antibacterial activity as 80% of isolates inhibited growth of *S. aureus*, 84% of isolates inhibited growth of *S. pyogenes*, and 8% isolates inhibited growth of *B. cereus*.

**Table-6:** *In vitro* antibacterial activity of endophytic bacteria isolated from *Azadirachta indica* (neem) against Gram-positive bacteria.

Isolate No.	*Staphylococcus aureus*	*Streptococcus pyogenes*	*Bacillus cereus*
N1a	S	S	R
N1b	S	S	R
N1c	S	R	R
N1d	S	S	R
N1e	R	S	R
N2a	S	S	S
N2b	S	S	R
N2c	S	S	R
N2d	S	S	R
N2e	S	R	R
N3a	R	S	R
N3b	S	S	R
N3c	S	S	R
N3d	S	S	R
N3e	S	S	S
N4a	S	R	R
N4b	S	S	R
N4c	R	S	R
N4d	S	S	R
N4e	R	S	R
N5a	S	S	R
N5b	S	S	R
N5c	R	R	R
N5d	S	S	R
N5e	S	S	R

#### For Gram-negative bacteria

The *in vitro* antibacterial activities of endophytic bacteria against different Gram-negative bacteria have been shown in Table-7. The endophytic bacteria isolated from *A. indica* (neem) shown antibacterial activity as 92% of isolates inhibited growth of *E. coli*, 88% of isolates inhibited growth of *Salmonella* Typhimurium, and 88% isolates inhibited growth of *K. pneumoniae*.

**Table-7:** *In vitro* antibacterial activity of endophytic bacteria isolated from *Azadirachta indica* (neem) against Gram-negative bacteria.

Isolate No.	*Escherichia coli*	*Salmonella* Typhimurium	*Klebsiella pneumoniae*
N1a	S	S	S
N1b	S	S	S
N1c	S	S	S
N1d	S	S	R
N1e	R	S	S
N2a	S	S	S
N2b	S	R	S
N2c	S	S	S
N2d	S	S	S
N2e	S	S	S
N3a	S	S	S
N3b	S	S	S
N3c	S	R	S
N3d	S	S	S
N3e	S	S	S
N4a	S	S	R
N4b	S	S	S
N4c	R	S	S
N4d	S	S	S
N4e	S	S	S
N5a	S	S	S
N5b	S	R	R
N5c	S	S	S
N5d	S	S	S
N5e	S	S	S

### Over all in vitro antibacterial activity of endophytic bacterial isolates

Out of 25 isolates from *A. indica* (neem) 20 isolates were effective against *S. aureus*, 21 against *S. pyogenes*, 2 against *B. cereus*, 23 against *E. coli*, 22 against *Salmonella* Typhimurium, and 22 against *K. pneumoniae* (Table-8).

**Table-8:** Overall *in vitro* antibacterial activity of endophytic bacterial isolates.

Samples	Activity against
N1a, N1b, N1c, N1d, N2a, N2b, N2c, N2d, N2e, N3b, N3c, N3d, N3e, N4a, N4b, N4d, N4e, N5b, N5d, N5e	*Staphylococcus aureus*
N1a, N1b, N1d, N1e, N2a, N2b, N2c, N2d, N3a, N3b, N3c, N3d, N3e, N4b, N4c, N4d, N4e, N5a, N5b, N5d, N5e	*Streptococcus pyogenes*
N2a, N3e	*Bacillus cereus*
N1a, N1b, N1c, N1d, N2a, N2b, N2c, N2d, N2e, N3a, N3b, N3c, N3d, N3e, N4a, N4b, N4d, N4e, N5a, N5b, N5c, N5d, N5e	*Escherichia coli*
N1a, N1b, N1c, N1d, N1e, N2a, N2c, N2d, N2e, N3a, N3b, N3d, N3e, N4a, N4b, N4c, N4d, N4e, N5a, N5c, N5d, N5e	*Salmonella* Typhimurium
N1a, N1b, N1c, N1e, N2a, N2b, N2c, N2d, N2e, N3a, N3b, N3c, N3d, N3e, N4b, N4c, N4d, N4e, N5a, N5c, N5d, N5e	*Klebsiella pneumoniae*

## Discussion

About 25 strains of endophytic bacteria were isolated from leaves of *A. indica* (neem). Endophytic bacteria are found in virtually every plant on earth [13]. Different plant parts such as root, stem and nodule [14], leaves, stems and root [15] can also be used for isolation of endophytic bacteria. Costa *et al*. [16] had isolated culturable endophytic bacteria from common bean (Phaseolus vulgaris) leaves.

The preliminary identification of the bacterial isolates was done based on various morphological features of isolated endophytic bacteria. The colony characteristics of endophytic bacteria isolated from neem are having irregular in shape while flat elevation on petri plate, margin of the colonies were undulated; the surface of the growth was rough, opaque and white in color. The microscopic examination of endophytic bacteria has shown that among the endophytic bacteria isolated from neem, 88% isolate were Gram-positive while 12% Gram-negative, 88% endophytic bacteria were rod shape and 12% were cocci, microscopic examination showed that there were more than one type of endophytic bacteria present in 84% of isolate.

The isolation of endophytic bacteria was in agreement with the findings of Hung and Annapurna [14] and had found equal percentages of Gram-positive (49%) and Gram-negative (51%) bacteria. Sobral *et al*. [15] and Ebrahimia *et al*. [17] have also found equal percentage of Gram-positive and Gram-negative bacteria. However, Bahgat *et al*. [18] found the 90% of Gram-positive bacteria.

As summarized in results antibacterial activity of endophytic bacteria was calculated by the presence of zone of inhibition produced by endophytic bacteria against pathogenic bacteria. All the isolates from endophytic bacteria were screened for the antibacterial activity against pathogenic bacteria *B. cereus, S. aureus, Streptococcus pyogenes, E. coli, K. pneumoniae*, and *Salmonella* Typhimurium.

The overall *in vitro* antibacterial results shown that maximum sensitivity was observed against *E. coli*. Most of the isolates from neem had shown antibacterial activity against both Gram-positive (*S. aureus*, and *S. pyogenes*) and Gram-negative bacteria (*E. coli, Salmonella* Typhimurium, and *K. pneumoniae*).

Verma *et al*. [19] observed antibacterial activity of endophytic actinomycetes from *A. indica* against *E. coli*. Ebrahimia *et al*. [17] observed antibacterial activity of endophytic bacteria isolated from leaves of *Hypericum scabrum* against *S. aureus*. Jalgaonwala *et al*. [20] observed antibacterial activity of endophytic bacteria isolated from roots of *Aloe vera* possess strong antibacterial activity against *S. typhi* in dual culture assay. Roy and Banerjee [21] isolated endophytic bacteria from a medicinal plant *Vinca rosea*. One of the isolated endophytes produced potential antimicrobial activity against *B. cereus, K. pneumoniae*, and *E. coli*. Pal *et al*. [22] reported the antimicrobial activity of the bacterial endophytes of *Passiflora foetida* indicating the inhibitory effect of the majority of the isolates against *E. coli, S. aureus*, and *K. pneumoniae*. This study is also very near to all the above authors.

## Conclusion

Endophytic bacteria were present in leaves of *A. indica* (neem), Gram-positive rod-shaped bacteria were present in leaves of *A. indica* (neem). Endophytic bacteria from neem possess maximum antibacterial activity against *S. aureus, S. pyogenes, E. coli, Salmonella* Typhimurium, *and K. pneumoniae*.

## Authors’ Contributions

AKS, RKS and VS planned and design the study and done the analysis of data. TS, RK and DK helped in the collection of samples and in the laboratory procedures during the research work. All authors read and approved the final manuscript.
